# 2-[(*Z*)-4,7-Dichloro-3,3-dimethyl-2,3-dihydro-1*H*-indol-2-yl­idene]-3-oxopropane­nitrile

**DOI:** 10.1107/S1600536811053906

**Published:** 2011-12-23

**Authors:** Madeleine Helliwell, Mehdi M. Baradarani, Razieh Mohammadnejadaghdam, Arash Afghan, John A. Joule

**Affiliations:** a The School of Chemistry, The University of Manchester, Manchester M13 9PL, England; b Department of Chemistry, Faculty of Science, University of Urmia, Urmia 57153-165, Iran; c Department of Chemical Engineering, University of Urmia, Urmia 57153-165, Iran

## Abstract

In the title compound, C_13_H_10_Cl_2_N_2_O, the ring N atom and its three attached atoms are essentially coplanar with angles adding to 359.8°, indicating conjugation with the 2-formyl­acrylonitrile subunit. The aldehyde group is oriented to place the carbonyl O atom 2.02 (3) Å from the N—H hydrogen atom. Intra­molecular N—H⋯O and C—H⋯Cl inter­actions occur. The geometry of the exocyclic double bond is *Z*. In the crystal, weak C—H⋯N hydrogen bonds link the mol­ecules into chains along [1
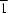
0].

## Related literature

For related structures, see: Baradarani *et al.* (2006 ▶); Helliwell *et al.* (2010 ▶) Rashidi *et al.* (2009 ▶). For the chemistry of complexes of  (2*H*-indol-2-ylidene)propanedials, see: Rashidi *et al.* (2011 ▶). 
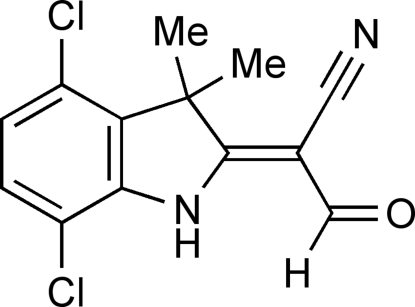


## Experimental

### 

#### Crystal data

C_13_H_10_Cl_2_N_2_O
*M* *_r_* = 281.13
Triclinic, 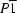

*a* = 7.0535 (8) Å
*b* = 7.9455 (10) Å
*c* = 12.2883 (15) Å
α = 105.151 (2)°
β = 104.855 (2)°
γ = 95.296 (2)°
*V* = 633.09 (13) Å^3^
*Z* = 2
Mo *K*α radiation
μ = 0.50 mm^−1^
*T* = 100 K
0.60 × 0.60 × 0.40 mm

#### Data collection

Bruker SMART CCD area-detector diffractometer
Absorption correction: multi-scan (*SADABS*; Bruker, 2001 ▶) *T*_min_ = 0.724, *T*_max_ = 1.000
3237 measured reflections
2268 independent reflections
2028 reflections with *I* > 2σ(*I*)
*R*_int_ = 0.036

#### Refinement

*R*[*F* ^2^ > 2σ(*F* ^2^)] = 0.046
*wR*(*F* ^2^) = 0.127
*S* = 1.05
2268 reflections
169 parameters
H atoms treated by a mixture of independent and constrained refinement
Δρ_max_ = 0.37 e Å^−3^
Δρ_min_ = −0.33 e Å^−3^

### 

Data collection: *SMART* (Bruker, 2001 ▶); cell refinement: *SAINT* (Bruker, 2002 ▶); data reduction: *SAINT*; program(s) used to solve structure: *SHELXS97* (Sheldrick, 2008 ▶); program(s) used to refine structure: *SHELXL97* (Sheldrick, 2008 ▶); molecular graphics: *SHELXTL* (Sheldrick, 2008 ▶); software used to prepare material for publication: *SHELXTL*.

## Supplementary Material

Crystal structure: contains datablock(s) global, I. DOI: 10.1107/S1600536811053906/zq2144sup1.cif

Structure factors: contains datablock(s) I. DOI: 10.1107/S1600536811053906/zq2144Isup2.hkl

Supplementary material file. DOI: 10.1107/S1600536811053906/zq2144Isup3.cml

Additional supplementary materials:  crystallographic information; 3D view; checkCIF report

## Figures and Tables

**Table 1:** Hydrogen-bond geometry (Å, °)

*D*—H⋯*A*	*D*—H	H⋯*A*	*D*⋯*A*	*D*—H⋯*A*
C3—H3⋯N2^i^	0.93	2.56	3.256 (3)	132
C10—H10*B*⋯Cl2	0.96	2.83	3.473 (2)	125
N1—H1*N*⋯O1	0.88 (3)	2.02 (3)	2.678 (3)	131 (2)

Symmetry code: (i) 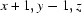.

**Fig. 1.**
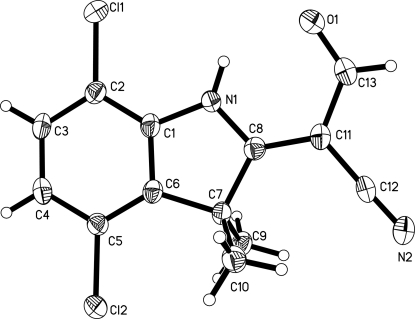
Plot of the title compound with ellipsoids drawn at the 50% probability level.

**Fig. 2.**
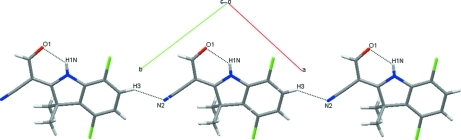
Packing diagram showing the intramolecular N—H···O hydrogen bonds and the weak intermolecular C—H···N hydrogen bonds, which link the molecules into chains.

**Fig. 3.**
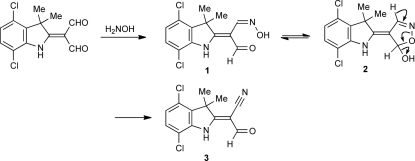
Reaction scheme.

## supplementary crystallographic information

### Comment

We showed that the interaction of 2,3,3-trimethyl-3*H*-indoles with the Vilsmeier reagent produces (1,3-dihydro-3,3-dimethyl-2*H*-indol-2-ylidene)propanedials (Baradarani *et al.*, 2006). 2,3,3-Trimethyl-2*H*-pyrrolo[2,3-*f*]quinoline, 2,3,3-trimethyl-3*H*-pyrrolo[3,2-*h*]quinoline (Rashidi *et al.*, 2009), 2,2',3,3,3',3'-hexamethyl-3*H*,3'*H*-5,5'-biindole and 2,3,3,7,8,8-hexamethyl-3*H*,8*H*-indolo[7,6-*g*]indole (Rashidi *et al.*, 2011) behave analogously. The (1,3-dihydroindol-2-ylidene)propanedials were shown to react with arylhydrazines (or hydrazine) to produce 3,3-dimethyl-2-[1-aryl-1*H*-pyrazol-4-yl]-3*H*-indoles (Baradarani *et al.*, 2006; Rashidi *et al.*, 2009; Helliwell *et al.* 2010; Rashidi *et al.*, 2011).

In anticipation that the (1,3-dihydroindol-2-ylidene)propanedials would react with hydroxylamine to produce isoxazol-4-yl-3*H*-indoles, 2-(4,7-dichloro-1,3-dihydro-3,3-dimethyl-2*H*-indol-2-ylidene)propanedial was treated with hydroxylamine hydrochloride in refluxing ethanol. The unexpected product of the reaction was 2-(4,7-dichloro-1,3-dihydro-3,3-dimethyl-2*H*-indol-2-ylidene)-3-oxopropanenitrile as shown by this X-ray diffraction analysis. We interpret this transformation as involving firstly formation of the monooxime 1 which cyclizes to generate hemiacetal 2, fragmentation of which (arrows on 2) would then give the product 3 (Fig. 3).

The sum of the angles of the bonds at the ring nitrogen in the title compound is 359.8 ° showing the extensive conjugation of the nitrogen with the 2-formylacrylonitrile subunit. The geometry of the double bond linking the two heterocyclic subunits is *Z*. In the crystal structure, there are intramolecular N—H···O and C—H···Cl interactions and weak intermolecular C—H···N hydrogen bonds which link the molecules into chains.

### Experimental

A mixture of 2-(4,7-dichloro-1,3-dihydro-3,3-dimethyl-2*H*-indol-2-ylidene)propanedial (100 mg, 0.35 mmol) and hydroxylamine hydrochloride (24 mg, 0.35 mmol) in absolute EtOH (10 ml) was heated at reflux for 12 h. The solvent was evaporated and resulting mixture dissolved in water and neutralized with aq. NaOH (2 N). The resulting precipitate was filtered off, washed with water, dried in air and recrystallized from EtOH. Yield 70%, mp 451–456 K, FT—IR (KBr) ν_max_ 3199, 2989, 2941, 2205, 1642, 1539, 1156, 928 cm^-1^, ^1^H NMR (CDCl_3_) δ 1.87 (s, 6H, 2CH_3_), 7.07 (d, *J* = 8.7 Hz, 1H, ArH), 7.25 (d, *J* = 8.7 Hz, 1H, ArH), 9.45 (s, 1H, CHO), 12.32 (bs, 1H, NH), ^13^C NMR (CDCl_3_) δ 20.3, 52.9, 81.6, 115.8, 117.8, 125.6, 127.1, 128.7, 129.7, 134.6, 139.4, 177.2, 188.0.

### Refinement

H atoms bonded to C were included in calculated positions using the riding method, with C—H distances of 0.96 Å and *U*_eq_ values set at 1.5 times those of the parent atoms for methyl H atoms and C—H distances of 0.93 Å and *U*_eq_ values of 1.2 times the parent atom for all other H atoms. The H atom bonded to N1 was found by difference Fourier methods and refined isotropically with the N1—H1N distance refined to 0.88 (3) Å.

### Crystal data

**Table:**

C_13_H_10_Cl_2_N_2_O	*Z* = 2
*M**_r_* = 281.13	*F*(000) = 288
Triclinic, *P*1	*D*_x_ = 1.475 Mg m^−^^3^
Hall symbol: -P 1	Mo *K*α radiation, λ = 0.71073 Å
*a* = 7.0535 (8) Å	Cell parameters from 954 reflections
*b* = 7.9455 (10) Å	θ = 2.7–26.6°
*c* = 12.2883 (15) Å	µ = 0.50 mm^−^^1^
α = 105.151 (2)°	*T* = 100 K
β = 104.855 (2)°	Irregular, colourless
γ = 95.296 (2)°	0.60 × 0.60 × 0.40 mm
*V* = 633.09 (13) Å^3^	

### Data collection

**Table:**

Bruker SMART CCD area-detector diffractometer	2268 independent reflections
Radiation source: fine-focus sealed tube	2028 reflections with *I* > 2σ(*I*)
graphite	*R*_int_ = 0.036
phi and ω scans	θ_max_ = 25.4°, θ_min_ = 1.8°
Absorption correction: multi-scan (*SADABS*; Bruker, 2001)	*h* = −7→8
*T*_min_ = 0.724, *T*_max_ = 1.000	*k* = −9→7
3237 measured reflections	*l* = −13→14

### Refinement

**Table:**

Refinement on *F*^2^	Primary atom site location: structure-invariant direct methods
Least-squares matrix: full	Secondary atom site location: difference Fourier map
*R*[*F*^2^ > 2σ(*F*^2^)] = 0.046	Hydrogen site location: inferred from neighbouring sites
*wR*(*F*^2^) = 0.127	H atoms treated by a mixture of independent and constrained refinement
*S* = 1.05	*w* = 1/[σ^2^(*F*_o_^2^) + (0.0661*P*)^2^ + 0.3121*P*] where *P* = (*F*_o_^2^ + 2*F*_c_^2^)/3
2268 reflections	(Δ/σ)_max_ < 0.001
169 parameters	Δρ_max_ = 0.37 e Å^−^^3^
0 restraints	Δρ_min_ = −0.33 e Å^−^^3^

### Special details

**Table:**

Geometry. All e.s.d.'s (except the e.s.d. in the dihedral angle between two l.s. planes) are estimated using the full covariance matrix. The cell e.s.d.'s are taken into account individually in the estimation of e.s.d.'s in distances, angles and torsion angles; correlations between e.s.d.'s in cell parameters are only used when they are defined by crystal symmetry. An approximate (isotropic) treatment of cell e.s.d.'s is used for estimating e.s.d.'s involving l.s. planes.
Refinement. Refinement of *F*^2^ against ALL reflections. The weighted *R*-factor *wR* and goodness of fit *S* are based on *F*^2^, conventional *R*-factors *R* are based on *F*, with *F* set to zero for negative *F*^2^. The threshold expression of *F*^2^ > σ(*F*^2^) is used only for calculating *R*-factors(gt) *etc*. and is not relevant to the choice of reflections for refinement. *R*-factors based on *F*^2^ are statistically about twice as large as those based on *F*, and *R*- factors based on ALL data will be even larger.

### Fractional atomic coordinates and isotropic or equivalent isotropic displacement parameters (Å^2^)

**Table:**

	*x*	*y*	*z*	*U*_iso_*/*U*_eq_	
Cl2	1.27782 (8)	0.70384 (7)	0.97251 (5)	0.0315 (2)	
Cl1	0.74564 (9)	0.06883 (7)	0.53899 (5)	0.0352 (2)	
O1	0.2509 (2)	0.4169 (2)	0.53396 (14)	0.0312 (4)	
N1	0.6292 (3)	0.4367 (2)	0.65761 (17)	0.0229 (4)	
N2	0.4289 (3)	1.0032 (3)	0.7934 (2)	0.0421 (6)	
C1	0.8201 (3)	0.3982 (3)	0.69974 (19)	0.0226 (5)	
C2	0.8935 (3)	0.2435 (3)	0.6587 (2)	0.0246 (5)	
C3	1.0883 (3)	0.2341 (3)	0.7155 (2)	0.0264 (5)	
H3	1.1416	0.1333	0.6900	0.032*	
C4	1.2042 (3)	0.3757 (3)	0.8108 (2)	0.0265 (5)	
H4	1.3336	0.3674	0.8489	0.032*	
C5	1.1276 (3)	0.5315 (3)	0.85021 (19)	0.0233 (5)	
C6	0.9348 (3)	0.5444 (3)	0.79314 (19)	0.0222 (5)	
C7	0.8125 (3)	0.6947 (3)	0.80972 (19)	0.0225 (5)	
C8	0.6153 (3)	0.6045 (3)	0.71454 (19)	0.0225 (5)	
C9	0.9066 (3)	0.8550 (3)	0.7818 (2)	0.0278 (5)	
H9A	0.9145	0.8194	0.7024	0.042*	
H9B	1.0379	0.8997	0.8353	0.042*	
H9C	0.8261	0.9459	0.7902	0.042*	
C10	0.7818 (4)	0.7484 (3)	0.9349 (2)	0.0298 (5)	
H10A	0.6960	0.8355	0.9390	0.045*	
H10B	0.9082	0.7970	0.9932	0.045*	
H10C	0.7224	0.6460	0.9494	0.045*	
C11	0.4428 (3)	0.6762 (3)	0.68668 (19)	0.0249 (5)	
C12	0.4373 (3)	0.8573 (3)	0.7470 (2)	0.0295 (5)	
C13	0.2650 (3)	0.5725 (3)	0.5954 (2)	0.0280 (5)	
H13	0.1531	0.6262	0.5818	0.034*	
H1N	0.528 (4)	0.371 (4)	0.599 (2)	0.033 (7)*	

### Atomic displacement parameters (Å^2^)

**Table:**

	*U*^11^	*U*^22^	*U*^33^	*U*^12^	*U*^13^	*U*^23^
Cl2	0.0233 (3)	0.0289 (3)	0.0350 (4)	0.0044 (2)	0.0003 (2)	0.0051 (2)
Cl1	0.0351 (4)	0.0219 (3)	0.0396 (4)	0.0050 (2)	0.0025 (3)	0.0021 (2)
O1	0.0254 (9)	0.0304 (9)	0.0331 (9)	0.0027 (7)	0.0033 (7)	0.0075 (7)
N1	0.0186 (9)	0.0207 (9)	0.0273 (10)	0.0042 (7)	0.0030 (8)	0.0070 (7)
N2	0.0358 (12)	0.0330 (12)	0.0495 (13)	0.0176 (9)	0.0022 (10)	0.0045 (10)
C1	0.0215 (11)	0.0220 (10)	0.0280 (11)	0.0062 (8)	0.0082 (9)	0.0117 (9)
C2	0.0260 (11)	0.0200 (10)	0.0283 (11)	0.0047 (9)	0.0077 (9)	0.0082 (9)
C3	0.0259 (12)	0.0230 (11)	0.0358 (12)	0.0108 (9)	0.0113 (10)	0.0132 (9)
C4	0.0213 (11)	0.0296 (11)	0.0341 (12)	0.0092 (9)	0.0085 (9)	0.0163 (9)
C5	0.0213 (11)	0.0223 (10)	0.0258 (11)	0.0032 (8)	0.0052 (9)	0.0081 (9)
C6	0.0226 (11)	0.0204 (10)	0.0266 (11)	0.0055 (8)	0.0086 (9)	0.0103 (8)
C7	0.0211 (11)	0.0205 (10)	0.0265 (11)	0.0068 (8)	0.0063 (9)	0.0073 (8)
C8	0.0227 (11)	0.0206 (10)	0.0259 (11)	0.0037 (8)	0.0081 (9)	0.0091 (8)
C9	0.0262 (12)	0.0201 (10)	0.0383 (13)	0.0054 (9)	0.0084 (10)	0.0110 (9)
C10	0.0266 (12)	0.0346 (12)	0.0278 (12)	0.0098 (10)	0.0071 (9)	0.0077 (9)
C11	0.0232 (11)	0.0259 (11)	0.0295 (12)	0.0087 (9)	0.0090 (9)	0.0119 (9)
C12	0.0211 (11)	0.0326 (13)	0.0338 (12)	0.0110 (9)	0.0035 (9)	0.0101 (10)
C13	0.0224 (11)	0.0342 (12)	0.0305 (12)	0.0078 (9)	0.0074 (9)	0.0143 (10)

### Geometric parameters (Å, °)

**Table:**

Cl2—C5	1.760 (2)	C6—C7	1.538 (3)
Cl1—C2	1.749 (2)	C7—C8	1.536 (3)
O1—C13	1.249 (3)	C7—C9	1.540 (3)
N1—C8	1.359 (3)	C7—C10	1.561 (3)
N1—C1	1.406 (3)	C8—C11	1.393 (3)
N1—H1N	0.88 (3)	C9—H9A	0.9600
N2—C12	1.165 (3)	C9—H9B	0.9600
C1—C2	1.400 (3)	C9—H9C	0.9600
C1—C6	1.405 (3)	C10—H10A	0.9600
C2—C3	1.392 (3)	C10—H10B	0.9600
C3—C4	1.399 (3)	C10—H10C	0.9600
C3—H3	0.9300	C11—C12	1.446 (3)
C4—C5	1.415 (3)	C11—C13	1.454 (3)
C4—H4	0.9300	C13—H13	0.9300
C5—C6	1.388 (3)		
			
C8—N1—C1	111.42 (18)	C6—C7—C10	112.45 (17)
C8—N1—H1N	119.7 (18)	C9—C7—C10	111.53 (18)
C1—N1—H1N	128.7 (18)	N1—C8—C11	122.6 (2)
C2—C1—C6	123.2 (2)	N1—C8—C7	110.02 (18)
C2—C1—N1	128.0 (2)	C11—C8—C7	127.41 (19)
C6—C1—N1	108.87 (18)	C7—C9—H9A	109.5
C3—C2—C1	117.9 (2)	C7—C9—H9B	109.5
C3—C2—Cl1	121.11 (17)	H9A—C9—H9B	109.5
C1—C2—Cl1	120.99 (17)	C7—C9—H9C	109.5
C2—C3—C4	120.1 (2)	H9A—C9—H9C	109.5
C2—C3—H3	119.9	H9B—C9—H9C	109.5
C4—C3—H3	119.9	C7—C10—H10A	109.5
C3—C4—C5	121.0 (2)	C7—C10—H10B	109.5
C3—C4—H4	119.5	H10A—C10—H10B	109.5
C5—C4—H4	119.5	C7—C10—H10C	109.5
C6—C5—C4	119.5 (2)	H10A—C10—H10C	109.5
C6—C5—Cl2	121.27 (16)	H10B—C10—H10C	109.5
C4—C5—Cl2	119.19 (17)	C8—C11—C12	120.6 (2)
C5—C6—C1	118.17 (19)	C8—C11—C13	121.2 (2)
C5—C6—C7	132.44 (19)	C12—C11—C13	118.26 (19)
C1—C6—C7	109.38 (18)	N2—C12—C11	178.4 (3)
C8—C7—C6	100.22 (16)	O1—C13—C11	125.0 (2)
C8—C7—C9	110.43 (18)	O1—C13—H13	117.5
C6—C7—C9	110.65 (17)	C11—C13—H13	117.5
C8—C7—C10	111.05 (18)		
			
C8—N1—C1—C2	−176.3 (2)	C1—C6—C7—C8	1.8 (2)
C8—N1—C1—C6	3.2 (2)	C5—C6—C7—C9	64.1 (3)
C6—C1—C2—C3	1.4 (3)	C1—C6—C7—C9	−114.8 (2)
N1—C1—C2—C3	−179.2 (2)	C5—C6—C7—C10	−61.3 (3)
C6—C1—C2—Cl1	−177.94 (16)	C1—C6—C7—C10	119.75 (19)
N1—C1—C2—Cl1	1.5 (3)	C1—N1—C8—C11	178.22 (19)
C1—C2—C3—C4	0.4 (3)	C1—N1—C8—C7	−2.0 (2)
Cl1—C2—C3—C4	179.71 (16)	C6—C7—C8—N1	0.1 (2)
C2—C3—C4—C5	−0.9 (3)	C9—C7—C8—N1	116.83 (19)
C3—C4—C5—C6	−0.3 (3)	C10—C7—C8—N1	−118.90 (19)
C3—C4—C5—Cl2	178.64 (17)	C6—C7—C8—C11	179.9 (2)
C4—C5—C6—C1	2.0 (3)	C9—C7—C8—C11	−63.4 (3)
Cl2—C5—C6—C1	−176.95 (15)	C10—C7—C8—C11	60.9 (3)
C4—C5—C6—C7	−176.9 (2)	N1—C8—C11—C12	−177.9 (2)
Cl2—C5—C6—C7	4.2 (3)	C7—C8—C11—C12	2.3 (3)
C2—C1—C6—C5	−2.6 (3)	N1—C8—C11—C13	1.2 (3)
N1—C1—C6—C5	177.89 (18)	C7—C8—C11—C13	−178.6 (2)
C2—C1—C6—C7	176.50 (19)	C8—C11—C13—O1	−1.5 (3)
N1—C1—C6—C7	−3.0 (2)	C12—C11—C13—O1	177.7 (2)
C5—C6—C7—C8	−179.3 (2)		

### Hydrogen-bond geometry (Å, °)

**Table:**

*D*—H···*A*	*D*—H	H···*A*	*D*···*A*	*D*—H···*A*
C3—H3···N2^i^	0.93	2.56	3.256 (3)	132.
C10—H10B···Cl2	0.96	2.83	3.473 (2)	125.
N1—H1N···O1	0.88 (3)	2.02 (3)	2.678 (3)	131 (2)

Symmetry codes: (i) *x*+1, *y*−1, *z*.
